# News and Notes

**Published:** 1911-01

**Authors:** 


### NEWS AND NOTES

### REGULAR MEETING OF FULTON COUNTY MEDICAL SOCIETY, NOVEMBER 3, 1910.

Reported by R. R. Daly, M. D.

Dr. Phinizy Calhoun read a paper on "Amaurosis Caused by Intestinal Toxemia."

Dr. Stirling said in most of these ases there is a retro-bulbar optic neuritis in which the vision may descend to nothing and yet the patients recover. The lesion is directly behind the eye and as a rule there is no visible evidence of its presence. The field of vision is often contracted and scotoma for color is often present. Hysteria simulates this disease. It is distinguished by throwing a strong light on the pupil and getting normal reaction, but when kept there a wobbling of the pupil occurs in retro-bulbar neuritis. Muscular imbalance is often associated with this trouble.

Dr. Stockard said that he had seen the case Dr. Calhoun reported and was sure the diagnosis was correct. He told of a case of convulsions which was cured by relieving the indican as in this case. He thinks every case should be treated on strictly a buttermilk diet.

Dr. Daly told of a case of excessive myopia. 14-D—in a young girl who had been able to prepare herself for college, but who could no longer see to study. Careful examination showed no change in refraction but indican and skatol were found in the urine. There was a high degree of intestinal putrefaction, which ultimately resulted in irritation about the eye great enough to interfere entirely with her work for a year. As to the treatment of indican, while it lay with the general practitioner or with the stomach specialist, it certainly could not depend upon diet alone. There must be extensive physical exercise to relieve the poisoned tissues of their burden and beside this care must be taken as to the injection of alcohol and other deleterious substances.

Dr. LeConte said that we are learning much in the last seven years. Intestinal putrefaction is very far-reaching in its effects. It is not well to depend too much upon the appearance of the indican, because pus in the urine will also show it. A microscopic examination should always be made. None of the articles spoke of the cause of indican. Really, it is only a symptom and a catarrhal colitis always pre-exists and permits the bacteria to develop. The majority of cases will take from ten to eighteen months to get well and so we cannot keep them on too strict a diet. A case of his had defective vision as a symptom of other troubles and also had Riggs' disease. When the teeth were cleansed the catarrhal colitis was relieved and the eye symptoms entirely disappeared.

Dr. Calhoun said, in closing, that he was glad to have brought out a discussion in this important matter.

Dr. Stirling read a paper on "Wounds of the Eye" which appears elsewhere in the Journal.

Dr. Calhoun said there was no more important subject to discuss in the way of injuries. He described a case of injury treated by a local doctor who gave simply boric acid as a wash. Two days later there was pus in the anterior chamber and ulcer in the cornea. The foreign body had not been removed. In

Birmingham, where, on account of the many mills and factories, the injuries to the eye are numerous, there are many different foreign bodies to be extracted. The companies require the cases to be seen regularly until all chance of inflammation has disappeared. It is not enough simply to remove the foreign body and let the patient go.

Dr. McRae told of a case of a wound in the anterior chamber resulting from a torpedo shell which struck the upper lid. On the second day the patient said he was getting blind, and there was slight hemorrhage of the retina, causing it to peel off. Now he can only distinguish objects after four years.

Dr. Stirling said, in closing, that the big companies employing men are very lax. Oftentimes some workman about the yards gains a little skill in taking foreign objects from the eye and he does this, always using some dirty method, often his tongue. The eyes become infected and thus vision is frequently lost.

---

### PROCEEDINGS OF THE AMERICAN PROCTOLOGICAL SOCIETY.

*(Continued.)*

### "A CASE OF LOCALIZED DERMATITIS FOLLOWING THE USE OF QUININE AND UREA AS A LOCAL ANESTHETIC IN A CASE OF FISSURE AND HEMORRHOIDS."

By Arthur Hebb, M. D., of Baltimore, Md.

These days after the use of a 1% aqueous solution of Quinine and Urea, as a local anesthetic in a case of fissure and hemorrhoids, erythema over the ischiorectal region developed followed by epidermolysis, then a profuse serous discharge which continued for four or five weeks. The wound showing little tendency to heal during this time.

### "A BRIEF REVIEW OF THE HISTORY OF THE AMERICAN PROCTOLOGICAL SOCIETY FROM ITS ORGANIZATION IN 1899 TO DATE."

READ BY THE SECRETARY OF THE SOCIETY, LEWIS H. ADLER, JR., M. D., PHILADELPHIA, PA.

The author believed the presentation of this paper would help to impress the profession with what had been accomplished, not alone for the individual profit and pleasure of the members of the organization, but likewise for the benefit of the profession as well as for the advancement of the science of medicine.

Attention was called to the fact that as the surgeon had rescued surgery from the "Society of Barbers and Bathers," and the obstetrician the practice of midwifery from the ignorant and often grossly careless midwife, so the proctologist, largely by means of this organization, has advanced diseases of the rectum and sigmoid from the domain of quackery to a recognized specialty, and has removed from the hands of the charlatan to a great extent, a fertile field for playing upon the credulity and ignorance of the populace.

The first effort to organize a National Proctological Association was made in June 1895, when the American Medical Association met in Baltimore. Dr. Samuel T. Earle at that time called together the following Proctologists to meet at his house:- Drs. James P. Tuttle, Samuel G. Gant, T. C. Martin, Joseph M. Mathews, and Leon Straus. The subject was talked over informally but no definite action was taken.

Including the present meeting at St. Louis, the Society will have convened twelve times. The respective meeting places, starting with the first session in 1899 at Columbus, Ohio, were, Washington, D. C.; St. Paul, Minnesota; Saratoga Springs, New York; New Orleans, Louisiana; Atlantic City, N. J.; Pittsburgh, Pa.; Boston, Mass.; Atlantic City, N. J.; Chicago, Ill.; Atlantic City, N. J.; and St. Louis, Mo.

The following Presidents, in the order named, have been, Drs. Joseph M. Mathews, James P. Tuttle, Thomas Chas. Martin, Samuel T. Earle, William M. Beach, J. Rawson Pennington,

Lewis H. Adler, Jr., Samuel G. Gant, A. Bennett Cooke, George B. Evans, Dwight H. Murray and George J. Cook.

Special attention was called to a paper by Dr. Wm. Bodenhamer, in 1903, upon "Atony of the Rectum," read by title, the author being ninety-four years of age.

The number of papers presented at the different meetings has varied from twelve to twenty-eight. The total number of articles read at all meetings, exclusive of the present session, have been one hundred and eighty-seven. The first year, a small volume of Transactions was issued. This, however, was discontinued until 1908, when it was decided to issue an annual volume for three consecutive years. Two have already been issued, and the remaining volumes will be published, following the present meeting. It seems to the writer that the publication of the Transactions annually should be made a permanent record of the work accomplished by the Society and do more to add dignity to and further the aims of the organization than anything else. Knowing that the Transactions will be published, the members will be stimulated to present papers at each meeting, and exert their best efforts in their preparation. Furthermore, the discussion of such papers, will be more energetically entered into and more carefully considered because of the knowledge that such discussion will be permanently recorded.

The membership began with thirteen and at the present time numbers thirty-seven active members. Five Fellows have resigned for various reasons and one has been transferred from the active to the honorary list. Nine honorary Fellows have been elected as follows:—Sir Charles B. Ball, Dublin, Ireland; Prof. Dr. Sonnenberg, Berlin, Germany; Dr. A. Tierlinck, Gand, Belgium; Mr. F. Swinford Edwards, London, England; Mr. W. W. Wallis, London, England; Mr. P. Lockhart Mummery, London, England, Mr. W. Ernest Miles, London, England; Dr. Edmund Andrews, Chicago, Ill.; Dr. Wm. Bodenhamer, New Rochelle, N. Y.,—the two later being now deceased.

The average attendance at the Annual Meetings has been over ninety-five per cent of the membership, a most excellent indication of the interest shown in the work of the organization.

In the body of the article reference was made to a number of important papers presented from the inception of the organization, to date, and from a cursory review of these varied papers selected for special mention, it will seem that the subject of Proctology in all its phrases has been amply covered at one time or another; many new instruments have been devised and exhibited; many original methods of operation have been suggested; and advanced methods of treatment introduced.

The discussion of the papers presented has been entered in to freely both by members and visitors. Criticisms, favorably or otherwise, have been made with impartiality, but never in the entire history of the organization has one word been uttered to impair the harmony of a fellowship which had endeared itself to its members and strengthened as the years have gone by. It is perhaps unusual in so large a body of men, working in the same field and with the same objects in view, not to find occasionally jealously or personal antagonism in some form or manner. The fact that this has happened in the history of the organization, is due perhaps, somewhat to the care exercised in the selection and election of its members, and the intimacy and close friendship existing between them.

Those interested in the Society's welfare cannot be but well satisfied with the result thus far obtained. But what of the future? Can the same interest be maintained, and if so, can it be along similar lines, or is it possible to awaken further interest by the introduction of some new procedures and ideas? Possibly the discussion of some one subject as the chief feature of the program, to be opened by one or more men, previously assigned different parts for consideration, to be followed by a general discussion, a suggestion which was made at the first session, but so far as the writer has been able to ascertain never carried out, might prove a means of increasing interest in the work of the organization.

*(To be Continued.)*

